# Syllabus of Lectures on Human Embryology

**Published:** 1894-11

**Authors:** 


Syllabus of Lectures on Human Embryology: An Introduction to the Study of Obstetrics and Gynæcology. For Medical Students and Practitioners. With a Glossary of Embryological Terms. By Walter Porter Manton, M.D., Professor of Clinical Gynæcology and Lecturer on Obstetrics in the Detroit College of Medicine; Fellow of the Royal Microscopical Society, of the British Zoological Society, American Microscopical Society, etc. Illustrated with seventy outline drawings and photo-engravings. 12mo, Cloth, 126 pages, interleaved for adding notes and other illustrations, $1.25 net. Philadelphia: The F. A. Davis Co., Publishers, 1914 and 1916 Cherry Street.

This little hand-book is an excellent means of memorizing the details of embryology.

It consists of a series of sections. Each section is divided into paragraphs. Each paragraph bears its title in heavy type and the paragraph is concise and clear in its statement of the particular subject indicated by the heading. Not only is it useful for students but can be advantageously used by the physician who is in active practice. The interleaving is, of course, highly useful to students. It is hardly necessory to say that the subjects treated in this book are of the first importance to every physician.

